# Supplementary figures and images for: Tau protein aggregation associated with SARS-CoV-2 main protease
Source: PLoS One. 2023 Aug 21;18(8):e0288138. doi: 10.1371/journal.pone.0288138 (PMC10441795; doi:10.1371/journal.pone.0288138)

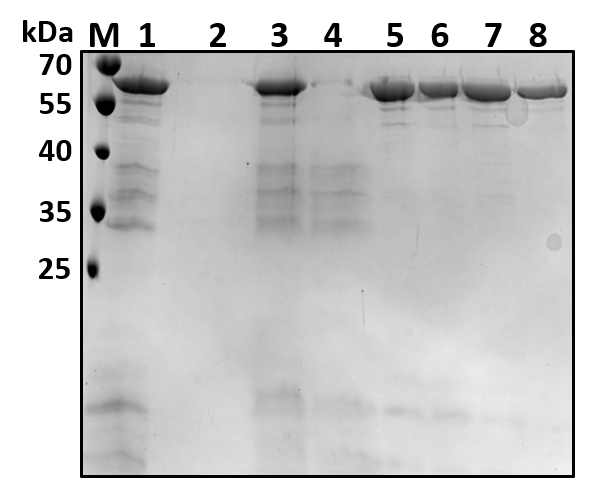

Supplement: S1 Fig — M: Protein marker; 1: Supernatant after cell disruption; 2: Supernatant after ammonium sulfate precipitation; 3: Pellet after ammonium sulfate precipitation solved in buffer 2; 4: SN after centrifugation pellet solved in buffer 2; 5: Pellet solved in ddH2O and 2 mM TCEP; 6: Pellet was solved in buffer 3; 7: SN after centrifugation pellet solved in buffer 3; 8: Sample after dialysis against buffer 4. (TIF) [file pone.0288138.s001.tif]

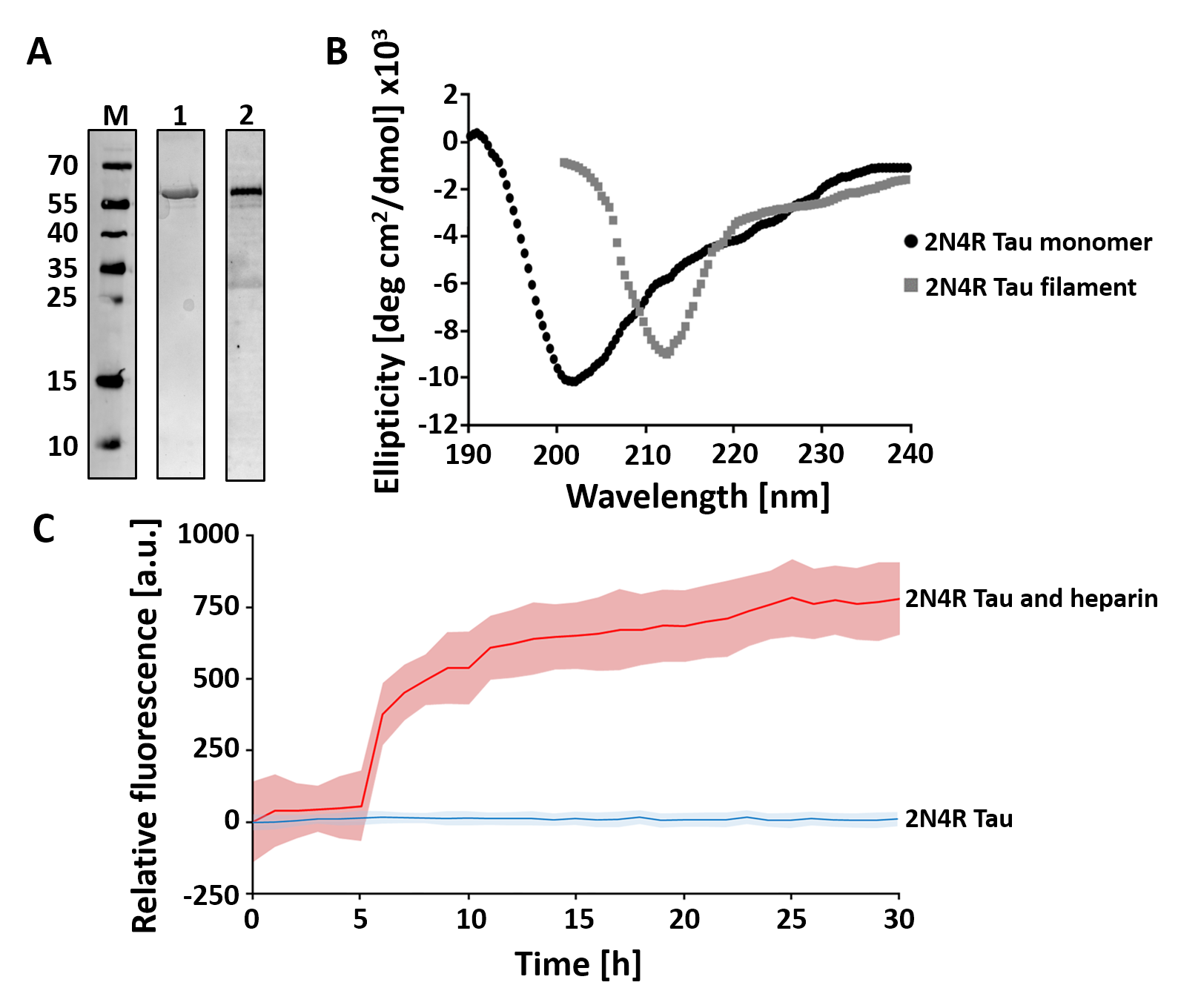

Supplement: S2 Fig — A: SDS PAGE 15% of 2N4R tau. M: protein marker, 1: pure 2N4R tau after precipitation purification, 2: western blot with CF633 labeled tau13. B: CD spectrum of 2N4R tau monomer and filament. C: ThT assay of 2N4R tau (10 μM), aggregation was induced with 2.5 μM heparin. Data shown are the mean ± SD from three independent measurements (n = 3). (TIF) [file pone.0288138.s002.tif]

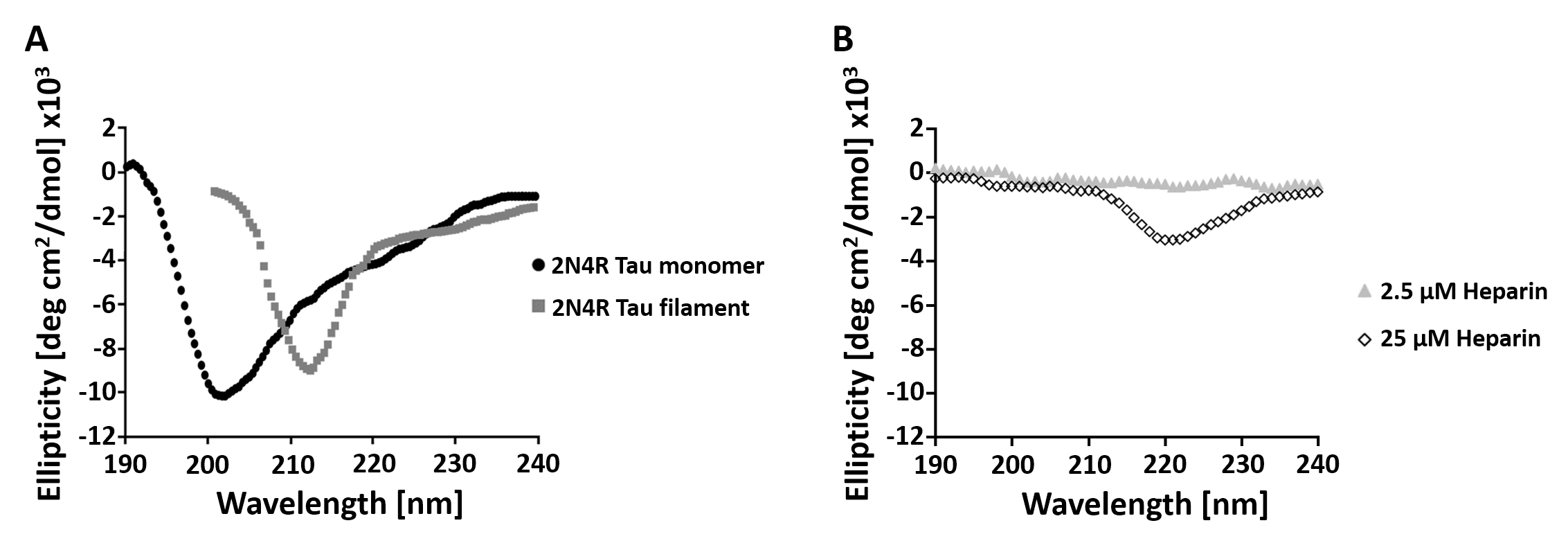

Supplement: S3 Fig — A: CD spectrum of 2N4R tau monomer and filament. B: CD spectrum of 2.5 μM and 25 μM heparin. (TIF) [file pone.0288138.s003.tif]

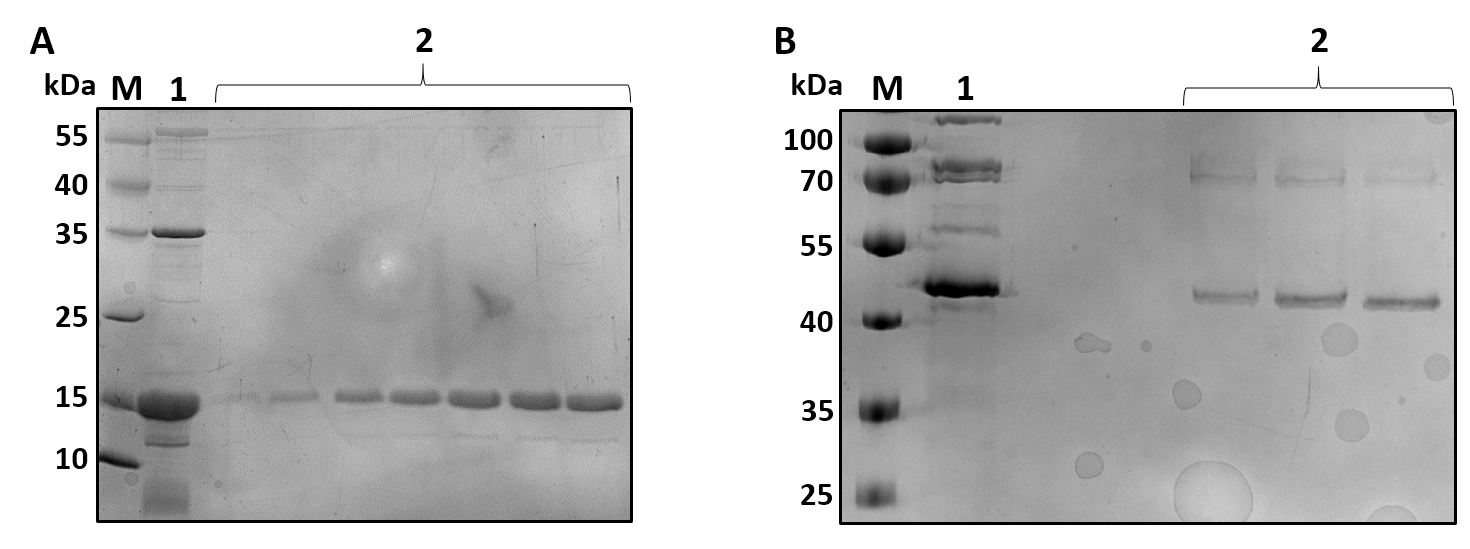

Supplement: S4 Fig — M: Protein marker; 1: Supernatant after affinity chromatography; 2: Elution steps after size exclusion chromatography. A: SDS PAGE of alpha-synuclein purification. B: SDS PAGE of TDP-43 purification. (TIF) [file pone.0288138.s004.tif]

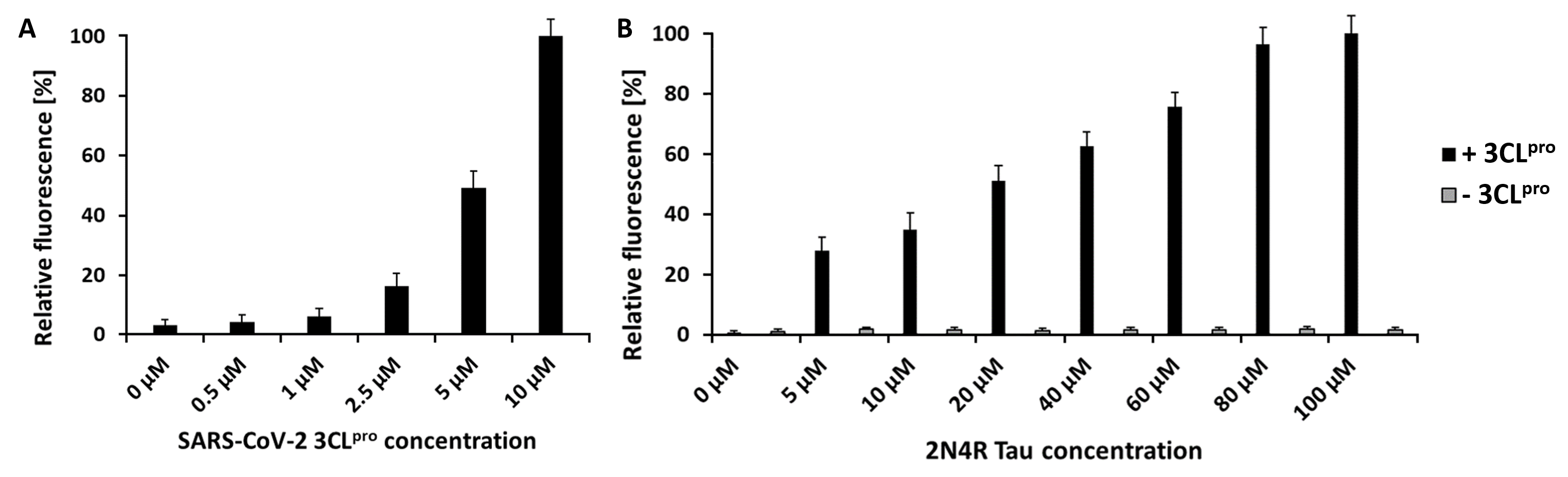

Supplement: S5 Fig — The endpoint of the relative fluorescence during a ThT assay is shown. A: Effect of different 3CLpro concentration (0, 0.5, 1, 2.5, 5 and 10 μM) on tau aggregation (Tau concentration was 10 μM). B: Effect of different tau concentration (0, 5, 10, 20, 40, 60, 80 and 100 μM) on tau aggregation. (3CLpro concentration was 10 μM). As Control experiments tau at each concentration without protease is shown. (TIF) [file pone.0288138.s005.tif]

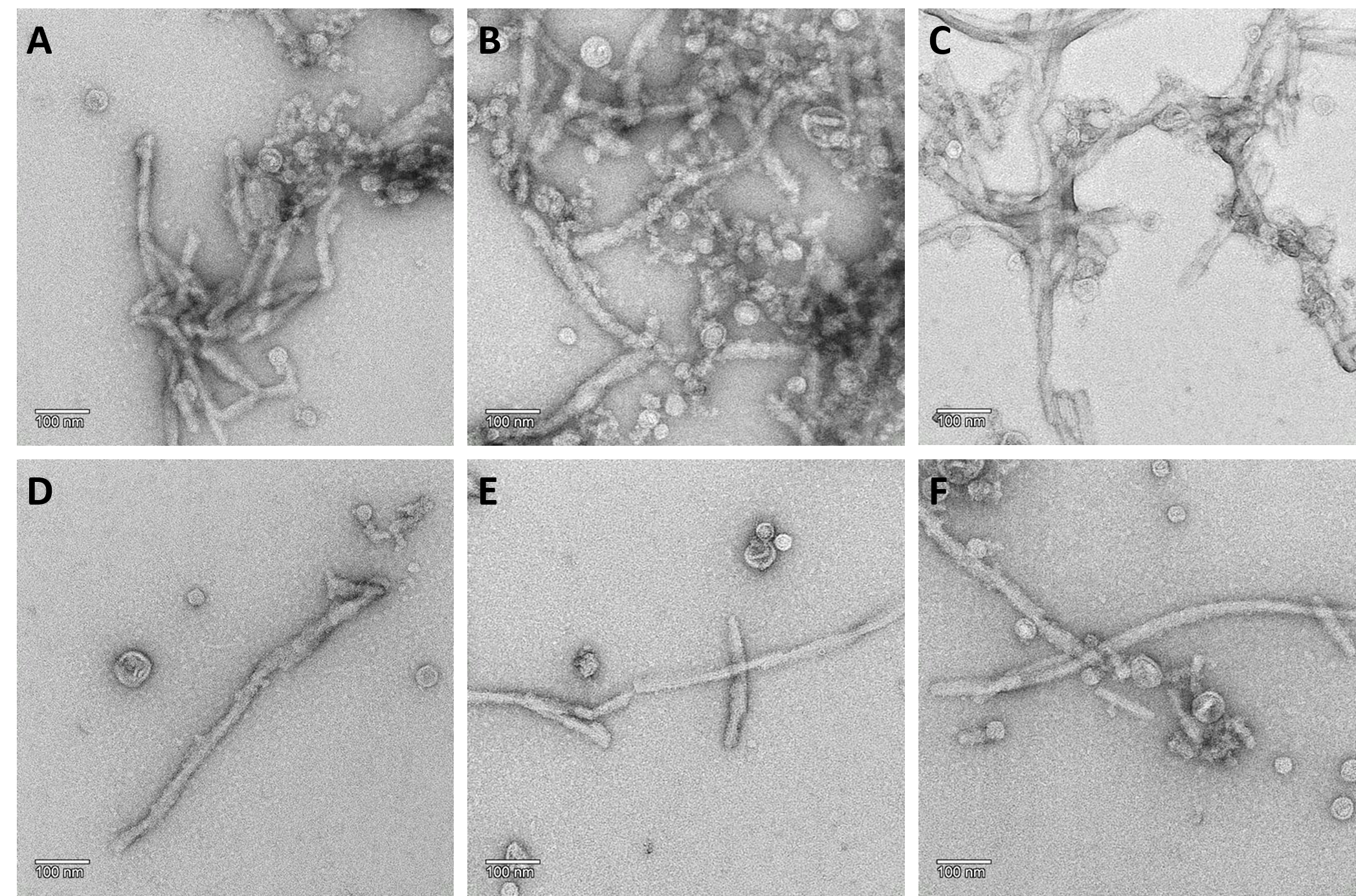

Supplement: S6 Fig — A-F: Representative micrographs at high (73kx) magnification, showing 3CLpro induced tau fibrilles. (TIF) [file pone.0288138.s006.tif]

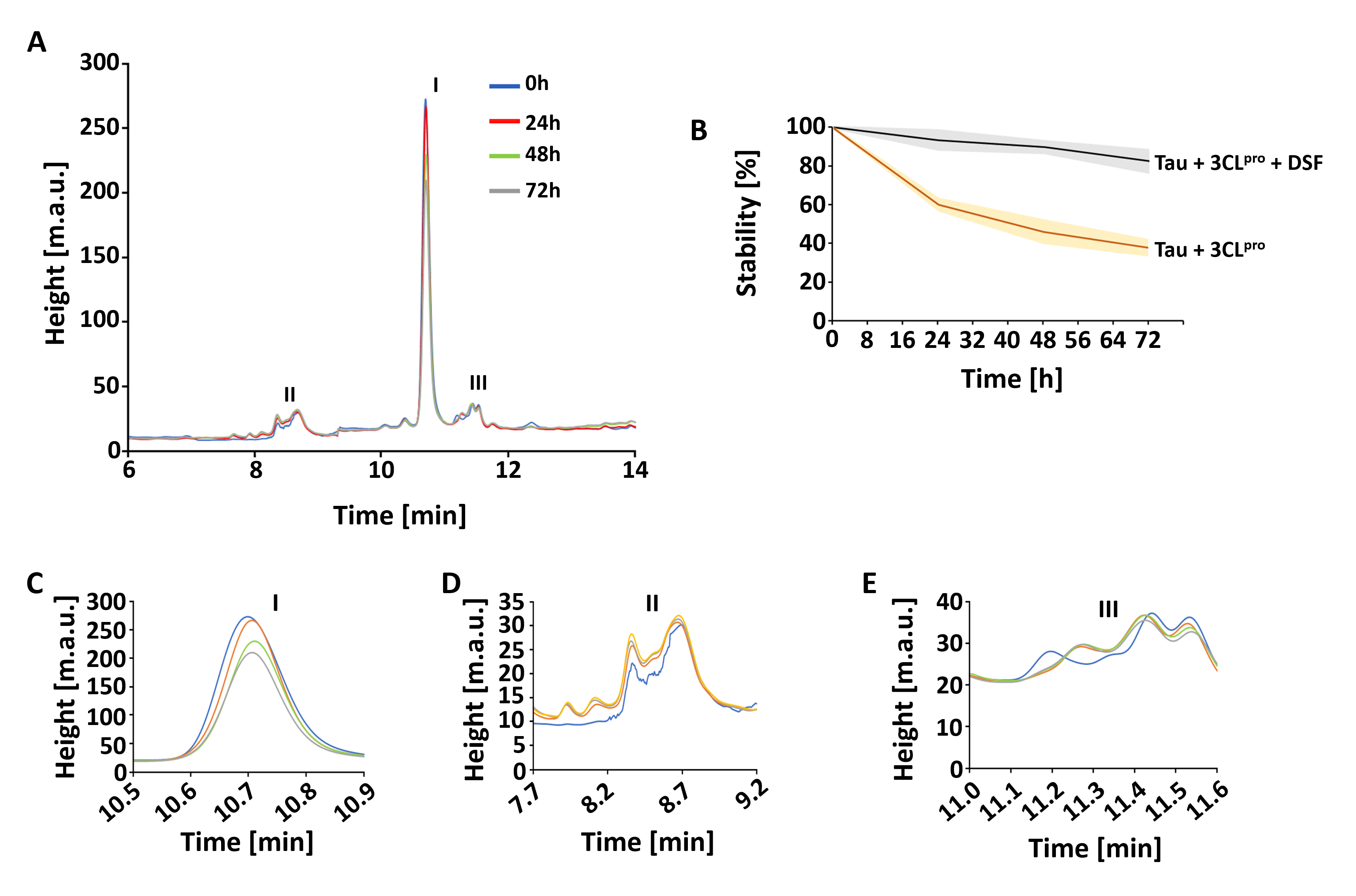

Supplement: S7 Fig — The protease was inactivated by 10 μM DSF. A: Analytical HPLC analysis of 2N4R tau incubated with inactivated SARS-CoV-2 3CLpro for 0, 24, 48 and 72h. The corresponding chromatogram regions of the tau monomer and related metabolites generated by active 3CLpro are highlighted (I-III). B: Stability of 2N4R tau monomer after treatment with inactivated 3CLpro over 72h. The tau monomer amount remains at 95%, compared with a control were tau was treated with active 3CLpro. In the control experiment the tau monomer amount reduce to around 40%. C: Chromatogram of peak I (Tau) shown enlarged, D: Chromatogram of peak region II shown and E: Chromatogram of peak region III shown enlarged. Data shown are the mean ± SD from three independent measurements (n = 3). (TIF) [file pone.0288138.s007.tif]

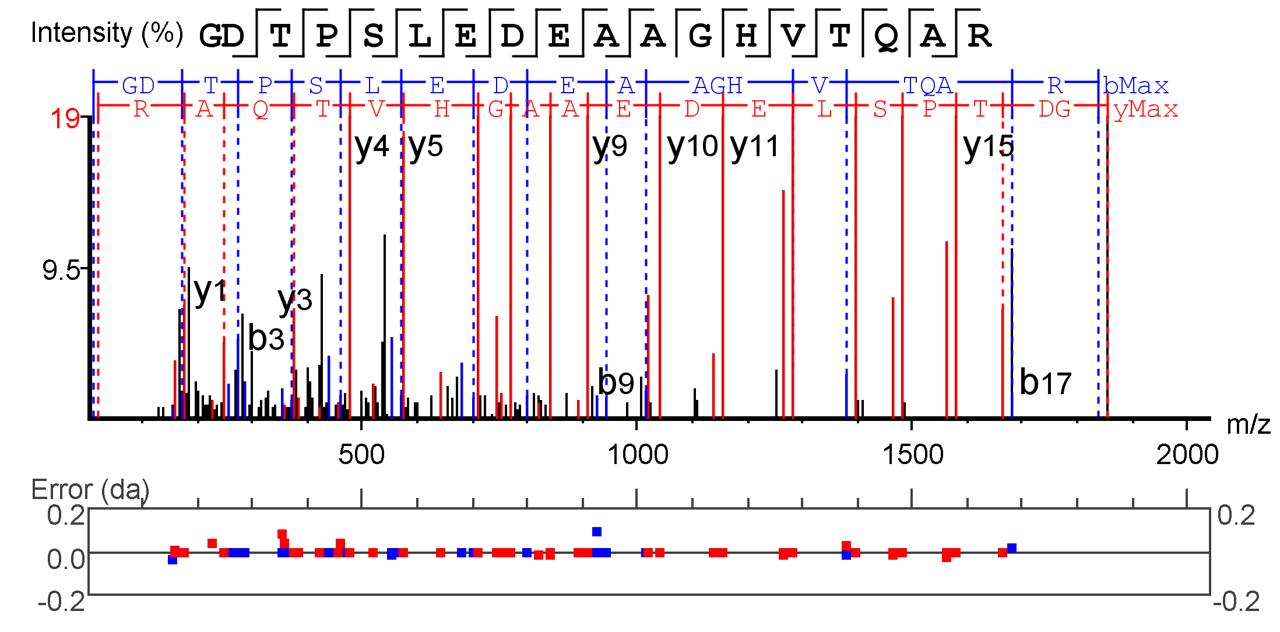

Supplement: S8 Fig — Example of a MS spectrum corresponding to a tryptic peptide derived from 2N4R tau (GDTPSLEDEAAGHVTQAR), b- and y-ions are labelled. (TIF) [file pone.0288138.s008.tif]

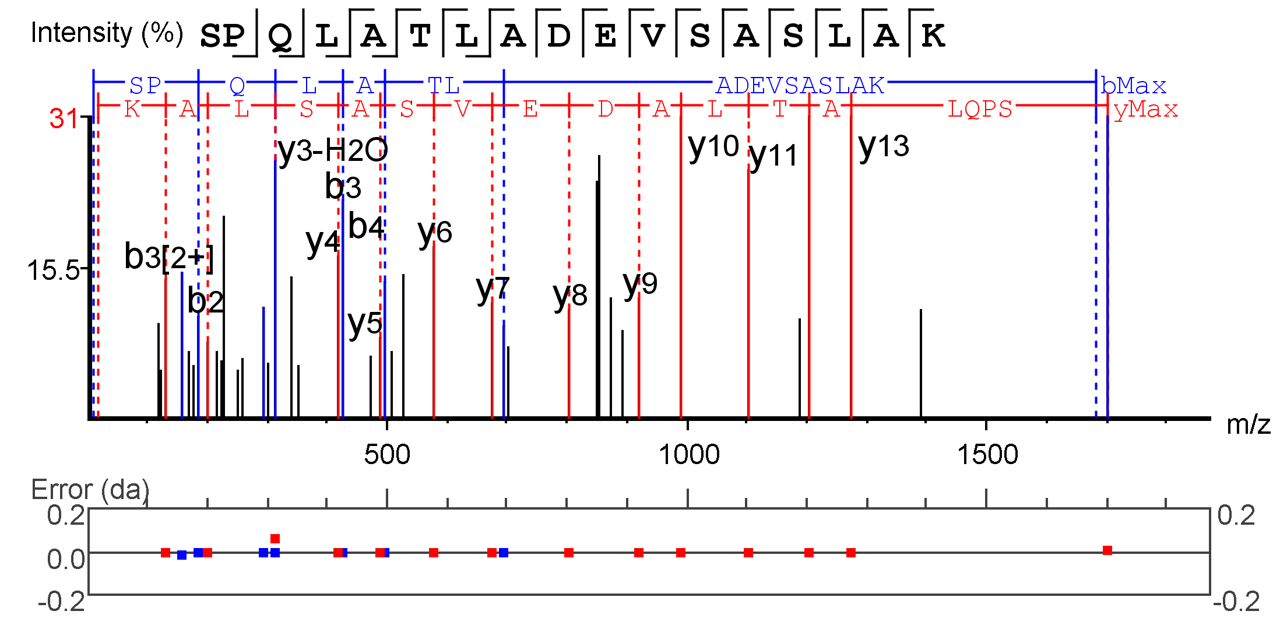

Supplement: S9 Fig — Example of a MS spectrum corresponding to a tryptic peptide derived from 2N4R tau (SPQLATLADEVSASLAK), b- and y-ions are labelled. (TIF) [file pone.0288138.s009.tif]

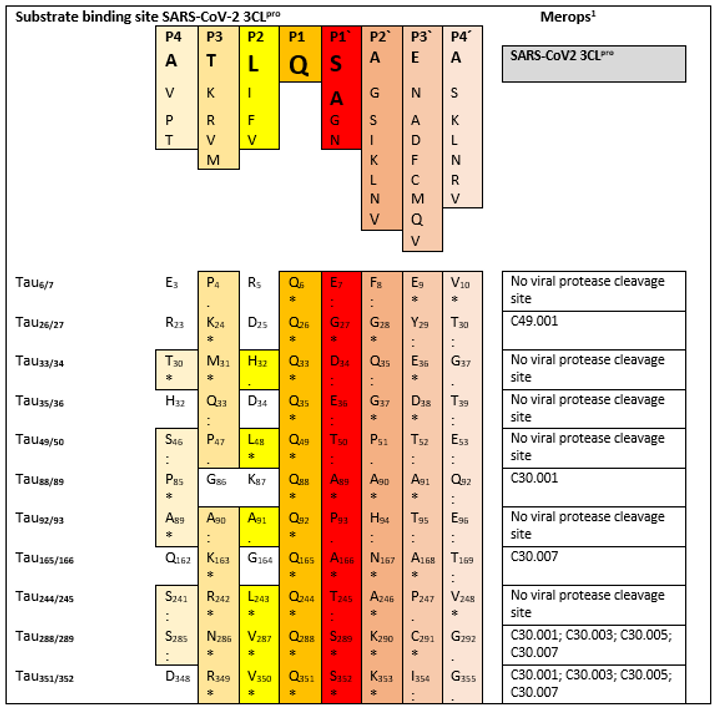

Supplement: S10 Fig — 3CLpro substrate binding site and preferred amino acids are shown and sequence homology of possible cleavage sites. The identical amino acid pattern was checked in the Merops database, if there exist identities to known viral protease cleavage sequences. The following virus proteases were identified: C49.001 (Strawberry mottle virus 3C-like peptidase); C30.001 (Coronavirus picornain 3C-like peptidase-1); C30.003 (Human coronavirus 229E main peptidase); C30.005 (SARS coronavirus picornain 3C-like peptidase) and C30.007 (Coronavirus COVID-19 3C-like peptidase). (TIF) [file pone.0288138.s010.tif]
