# Supplementary material for: Tau protein aggregation associated with SARS-CoV-2 main protease
Source: PLoS One. 2023 Aug 21;18(8):e0288138. doi: 10.1371/journal.pone.0288138 (PMC10441795; doi:10.1371/journal.pone.0288138)
Supplement: S1 Table — (PDF) [file pone.0288138.s011.pdf]

Table S1. Tryptic peptides of 2N4R tau.

	2N4R tau	m/z	ppm	length	Mass	Feature	Accession
1	AEPRQEFEVMEDHAGTYGLGDR	8363807	9	22	25061182	8	Human_TAU
2	GIGDTPSLEDEAAGHVTQAR	10124886	11	20	20229606	7	Human_TAU
3	HVPGGGSVQIVY	6068225	5	12	12116299	7	Human_TAU
4	GDTPSLEDEAAGHVTQAR	9274343	-6	18	18528551	6	Human_TAU
5	TPSLEDEAAGHVTQAR	8414106	0	16	16808066	6	Human_TAU
6	AGLKESPLQTPTEDGSEEPGSETSDAK	13806379	-2	27	27592620	5	Human_TAU
7	TAPVPMPDLK	5347914	-3	10	10675685	5	Human_TAU
8	TDAGLKESPLQTPTEDGSEEPGSETSDAK	14886765	6	29	29753367	4	Human_TAU
9	AEPRQEFEVMEDHAGTYGLGDRK	8790784	1	23	26342131	4	Human_TAU
10	STPTAEDVTAPLVDEGAPGKQ	10420134	3	21	20820117	4	Human_TAU
11	SLDNITHVPGGGNK	7048621	-7	14	14077106	4	Human_TAU
12	SPQLATLADEVSASLAKQGL	10000389	0	20	19980632	4	Human_TAU
13	AEPRQEFEVMEDHAGTY	10049391	2	17	20078632	4	Human_TAU
14	PSLPTPPTREPK	4405823	4	12	13187245	4	Human_TAU
15	PRQEFEVMEDHAGTYGLGDR	7696855	-16	20	23060386	3	Human_TAU
16	TPPSSGEPPKSGDRSGYSSPGSPGTPGSR	9294379	5	29	27852903	3	Human_TAU
17	TDHGAEIVYKSPVVSGD	8874371	9	17	17728580	3	Human_TAU
18	SKIGSLDNITHVPGGGNK	8974785	-4	18	17929431	3	Human_TAU
19	GSLGNIHHKPGGGQVEVK	9074884	15	18	18129595	3	Human_TAU
20	IGSLDNITHVPGGGN	7258679	1	15	14497212	3	Human_TAU
21	QTAPVPMPDLK	5988204	-7	11	11956271	3	Human_TAU
22	QIVYKPVDLSK	6453762	-9	11	12887390	3	Human_TAU
23	IVYKPVDLSK	5813475	0	10	11606804	3	Human_TAU
24	PVPMPDLK	4487482	-21	8	8954837	3	Human_TAU
25	SLPTPPTREPK	6118436	8	11	12216716	3	Human_TAU
26	QEFEVMEDHAGTY	7783214	-9	13	15546296	3	Human_TAU
27	PTPPTREPK	3415256	-7	9	10215556	3	Human_TAU
