# Supplementary material for: Tau protein aggregation associated with SARS-CoV-2 main protease
Source: PLoS One. 2023 Aug 21;18(8):e0288138. doi: 10.1371/journal.pone.0288138 (PMC10441795; doi:10.1371/journal.pone.0288138)
Supplement: S2 Table — (PDF) [file pone.0288138.s012.pdf]

Table S2. Tryptic peptides of 2N4R tau detected in peak I.

	Peak I	m/z	ppm	length	Mass	Feature	Accession
1	SPQLATLADEVSASLAK	8509570	2	17	16998992	8	Human_TAU
2	GDTPSLEDEAAGHVTQAR	9274293	-59	18	18528551	5	Human_TAU
3	PTPPTREPK	5117853	4	9	10215556	5	Human_TAU
4	AGLKESPLQTPTEDGSEEPGSETSDAK	9207599	-14	27	27592620	4	Human_TAU
5	PTAEDVTAPLVDEGAPGK	8839431	-10	18	17658733	4	Human_TAU
6	TPSLEDEAAGHVTQAR	8414100	-7	16	16808066	4	Human_TAU
7	GIGDTPSLEDEAAGHVTQAR	10124829	-46	20	20229606	4	Human_TAU
8	SPQLATLADEVSASLAKQGL	10000392	3	20	19980632	4	Human_TAU
9	AEPRQEFEVMEDHAGTYGLGDR	8363809	10	22	25061182	3	Human_TAU
10	DTPSLEDEAAGHVTQAR	8989072	-177	17	17958336	3	Human_TAU
11	SLDNITHVPGGGNK	7048618	-11	14	14077106	3	Human_TAU
12	VQIVYKPVDLSK	4636093	-11	12	13878075	3	Human_TAU
13	PSLPTPPTREPK	4405822	2	12	13187245	3	Human_TAU
14	IVYKPVDLSK	5813471	-7	10	11606804	3	Human_TAU
15	SLPTPPTREPK	6118428	-5	11	12216716	3	Human_TAU
