# Supplementary material for: Tau protein aggregation associated with SARS-CoV-2 main protease
Source: PLoS One. 2023 Aug 21;18(8):e0288138. doi: 10.1371/journal.pone.0288138 (PMC10441795; doi:10.1371/journal.pone.0288138)
Supplement: S3 Table — (PDF) [file pone.0288138.s013.pdf]

Table S3. Tryptic peptides of 2N4R tau detected in peak region II.

	**Peak region II**	**m/z**	**ppm**	**length**	**Mass**	**Feature**	**Accession**
1	GDTPSLEDEAAGHVTQAR	9274330	-20	18	18528551	6	Human_TAU
2	GIGDTPSLEDEAAGHVTQAR	10124861	-14	20	20229606	4	Human_TAU
3	STPTAEDVTAPLVDEGAPGKQ	10420308	169	21	20820117	4	Human_TAU
4	SPQLATLADEVSASLAK	8509573	5	17	16998992	4	Human_TAU
5	PTAEDVTAPLVDEGAPGK	8839406	-38	18	17658733	3	Human_TAU
6	LATLADEVSASLAK	6948846	-8	14	13877559	3	Human_TAU
7	SLDNITHVPGGGNK	7048621	-7	14	14077106	3	Human_TAU
8	HVPGGGSVQIVY	6068227	8	12	12116299	3	Human_TAU
9	GNIHHKPGGGQVEVK	5196147	2	15	15558219	3	Human_TAU
10	GSVQIVYKPVDLSK	7669375	-3	14	15318610	3	Human_TAU
11	PTPPTREPK	5117839	-22	9	10215556	3	Human_TAU
