# Supplementary material for: Tau protein aggregation associated with SARS-CoV-2 main protease
Source: PLoS One. 2023 Aug 21;18(8):e0288138. doi: 10.1371/journal.pone.0288138 (PMC10441795; doi:10.1371/journal.pone.0288138)
Supplement: S4 Table — (PDF) [file pone.0288138.s014.pdf]

Table S4. Tryptic peptides of 2N4R tau detected in peak region III.

	Peak region III	m/z	ppm	length	Mass	Feature	Accession
1	SPQLATLADEVSASLAK	5676406	5	17	16998992	15	Human_TAU
2	SLGNIHHKPGGGQVEVK	8789772	10	17	17559380	4	Human_TAU
3	SLDNITHVPGGGNK	7048634	12	14	14077106	4	Human_TAU
4	SLDNITHVPGGGNKK	5129423	-3	15	15358055	4	Human_TAU
5	GDTPSLEDEAAGHVTQAR	9274317	-34	18	18528551	3	Human_TAU
6	SNVSSTGSIDMVDSPQLATLADEVSASLAK	9984936	15	30	29924546	3	Human_TAU
7	GIGDTPSLEDEAAGHVTQAR	10124868	-8	20	20229606	3	Human_TAU
8	SPQLATLADEVSASLAKQGL	10000397	8	20	19980632	3	Human_TAU
9	PGGGSVQIVYKPVDLSK	8724852	-4	17	17429567	3	Human_TAU
10	DTPSLEDEAAGHVTQAR	8989229	-13	17	17958336	3	Human_TAU
11	IGSLDNITHVPGGGN	7258684	7	15	14497212	3	Human_TAU
12	GGGSVQIVYKPVDLSK	8239599	9	16	16459038	3	Human_TAU
13	QIVYKPVDLSK	6453778	16	11	12887390	3	Human_TAU
14	VYKPVDLSK	5248058	6	9	10475964	3	Human_TAU
15	SLPTPPTREPK	6118428	-5	11	12216716	3	Human_TAU
16	PTPPTREPK	5117839	-24	9	10215556	3	Human_TAU
